# Supplementary material for: Exploring the spatiotemporal dynamics and resilience assessment of urban networks from the perspective of population flow
Source: PLoS One. 2025 Jun 18;20(6):e0325908. doi: 10.1371/journal.pone.0325908 (PMC12176158; doi:10.1371/journal.pone.0325908)
Supplement: S1 Data — (ZIP) [file pone.0325908.s001.zip › Data File and Availability Statement/Data Availability Statement.docx]

**Data Availability Statement**

The data come from the Baidu Migration Platform (https://qianxi.baidu.com), a platform for public use and access to big data. In this study, the daily migration scale index of each city in the study area in one year and the ratio of the scale of inbound and outbound migration between cities were collected from this platform as the basic data, and the results of the study were obtained after calculation and processing.

This study does not involve any human experiment. The data of the study does not contain name, initials, physical address, internet protocol address, specific dates (birth dates, death dates, examination dates, etc.), contact information, or location data. The data used in the study does not have identifiers, does not involve specific groups, and ensures that no information on the identity of the population will be identified. The characteristics of this data are as follows:

1. Data desensitization: Standardized and processed migration scale indices and proportions are used as research indicators. The values are relative only and do not contain absolute units or orders of magnitude, to avoid exposing the details of the original data.

2. Spatial generalization: The starting point of the migration trajectory takes “city” as the smallest unit (population size greater than 1 million as the smallest unit), and does not involve specific address or location information. For example, the administrative boundaries of cities such as Shanghai and Nanjing are used as statistical units.

3. Time aggregation: the raw data are processed according to the daily average, eliminating hourly fluctuation characteristics, and further weakening the individual recognition of the time dimension. All processed data are not associated with the privacy of individuals or specific groups.

In summary, the data in this study meet the requirements of *PLOS One* and relevant laws and regulations.
