# Supplementary material for: Exploring the spatiotemporal dynamics and resilience assessment of urban networks from the perspective of population flow
Source: PLoS One. 2025 Jun 18;20(6):e0325908. doi: 10.1371/journal.pone.0325908 (PMC12176158; doi:10.1371/journal.pone.0325908)
Supplement: Attachment — Submitted filename: Response to Reviewers.docx [file pone.0325908.s002.docx]

**Response to Reviewers**

Dear Editors and Reviewers:

We sincerely appreciate your constructive feedback on our manuscript entitled “*Exploring the Spatiotemporal Dynamics and Resilience Assessment of Urban Networks from the Perspective of Population Flow*” (Manuscript Number: PONE-D-25-01272). Your expert comments have provided invaluable guidance for enhancing both the scholarly rigor and practical relevance of this study.

We have meticulously addressed each suggestion through comprehensive revisions. Accordingly, our point-by-point responses are organized as follows:

(1) **Response to Reviewer #1: Pages 2-4**.

(2) **Response to Reviewer #2: Pages 5-11**.

(3) **Response to Reviewer #3: Pages 12-17**.

Thank you for your time and thoughtful consideration of our work.

Best regards,

Sincerely,

Jiulin Li，Wenhui Lin，Jinlong Chu

**Response to Reviewer #1**

*This manuscript explores the spatiotemporal dynamics and resilience assessment of urban networks in the Yangtze River Delta region, based on complex network theory. The topic is of significant relevance, and the methodology is generally sound. However, there are a few areas that need further clarification to enhance the scientific rigor and credibility of the paper. The specific review comments are as follows:
1. Clarification on the Origin and Calculation of Network Metrics: In lines 229-268, several network metrics are introduced, including clustering coefficient, average path length, network efficiency, average degree of the network, and relative size of the largest connected subgraphs. However, the paper does not provide citations for the origin of these metrics. If these metrics are proposed by the authors, it is important to elaborate on their calculation methods and provide theoretical justification to demonstrate their credibility and relevance for assessing network efficiency.*

**The author’s answer:** Thank you for your valuable suggestions. The relevant metrics were derived from classic literature and were previously cited improperly. The revised manuscript now includes detailed annotations and citations for each metric. For more information, please refer to **"2.4 Methods" and "References"** in the manuscript.

*2. Inconsistency between Table 2's Title and Content: The title of Table 2 is “Top 25 city pairs in terms of intensity of population flow during the Spring Festival and daily period,” but the table only lists 10 pairs of cities for each of the two periods. The title should be revised to accurately reflect the content of the table, or the authors should explain why only 10 pairs are included.*

**The author’s answer:** Thank you for your careful review. We apologize for the previous inaccuracy. After carefully checking the raw data and manuscript content, We have revised the title of Table 2 to “**Top 10 city pairs** in terms of intensity of population flow during the Spring Festival and daily period.”

*3. Unclear Description of “Intentional Perturbation”: In section 3.2.1, the term “intentional perturbation” is mentioned, but its precise meaning and application are unclear. The paper does not specify which cities were targeted by the perturbation or provide any details on how the perturbation was implemented. It would be helpful to clarify the definition of “intentional perturbation” and provide further explanation of its implementation and impact, so that readers can better understand the research design.*

**The author’s answer:** Thank you for your valuable suggestions. We apologize for the lack of specificity in the previous formulation of the disturbance scenario. Based on the research context and field practice, **the English expression of “intentional perturbation” is revised to “targeted disruption”** to clarify the goal-oriented nature of the disturbance. After the revision, the terminology of the whole article is unified to avoid ambiguity.

In the revised article, **“2.4.2 Methods of scenario simulation”** is added in the “2.4 Methods” section, which illustrates the simulation logic of normal and disruption scenarios. Please refer to **“(2) Disruption scenario: dynamic characteristics analysis of network resilience”** for explanations of the conceptual connotation, implementation method, measurement indices and their roles of random and targeted disruptions. The following table is a brief description of the random disruption and targeted disruption in the scenario simulation. Please see **part (2) in section 2.4.2** of the paper for further details.

Table. Brief description of random and targeted disruptions

| **Disruption scenario** | **Academic definition** | **Scientific significance** | **Real-world example** |
| --- | --- | --- | --- |
| Random disruption | Randomly remove network nodes and associated edges with uniform probability to simulate unpredictable sudden shocks. | Evaluate the resilience change of networks to random failures. | Random interruption of road traffic caused by an earthquake. |
| Targeted disruption | Remove nodes and associated edges in descending order of importance to simulate intentional attacks. | Identify core network nodes and cascading failure risks. | Precision strikes on hub cities during wartime. |

*Overall, the manuscript presents a valuable analysis framework, but additional details and clearer references are needed to enhance the scientific rigor of the paper.*

**The author’s answer:** We extend our heartfelt gratitude for your thorough review and insightful comments, which have significantly improved the study. We have addressed each of your suggestions and revised the manuscript accordingly. Your emphasis on scientific rigor has deepened our appreciation for precision in research, and we will carry these lessons forward as valuable guidance for our future scholarly endeavors.

**Response to Reviewer #2**

*Many studies have used population flow data to construct urban networks and analyze their structure and resilience characteristics. This article is not innovative enough in terms of methodological design, selection of network structure indicators and resilience measurement, and it does not draw interesting or outstanding results. In addition, I don't see “The static characteristics of network resilience in natural scenarios were analyzed” mentioned by the author in the abstract. The authors just analyzed the structural characteristics of networks using some common network metrics. Specifically, the article has the following deficiencies:
1. There are some problems in the structural arrangement of the article. Usually, the authors should first further discuss the main findings of the study in the discussion section, such as comparing their own research methods, contents and conclusions with the current study, so as to better reflect the innovation of the article. Then in the final section of conclusion, the authors should summarize the methodology, conclusions, research significance, policy implications, and outlook for future research.*

**The author’s answer:** We greatly appreciate your insightful comments and suggestions. The contents of sections "4 Discussion" and "5 Conclusion" have been thoroughly revised according to the comments. Specifically, this includes: (1) Section **"4.1 Research significance and characteristics of population flow network resilience"** clarifies the main findings of this study. It also compares the research methodology, content, and conclusions with the current research, and identifies the similarities and differences, as well as the reasons for them. The theoretical significance of this study in the design of the theoretical framework and the positive impact on reality are explained. (2) Referring to the common structure of the papers published in *PLOS One*, the manuscript also writes **"4.2 Policy implications"** and **"4.3 Limitations and future directions"** in the discussion. (3) In **"5 Conclusion"**, the research methodology and significance of this study will be condensed, and the conclusion will be summarized more comprehensively.

**Reasons for choosing population mobility data for this study and possible innovations:** (1) Population, as a key carrier of all kinds of mobility factors such as economy, transportation and culture, has always been an important basis for studying the dynamic development of cities and society. At present, despite the slowing trend of global population growth, the increase of mobile population in some regions continues to rise. As a globally important economic growth pole, Jiangsu, Zhejiang and Shanghai have long ranked among the top regions in China in terms of mobile population size, which makes the region an ideal sample for studying the structural characteristics and resilience response mechanisms of urban networks. Therefore, although there are existing studies that use population mobility data to conduct relevant analyses, the perspective of this study is still of unique value. (2) This study fully absorbs and integrates the existing research methods, constructs the research framework of "structure measurement-scenario simulation-resilience assessment", and systematically reveals the resilience response mechanism of urban networks through static and dynamic scenario simulation. This provides a meaningful research paradigm for urban network resilience research. (3) Based on the urban network resilience assessment results, a "resilience-oriented" regional policy design path is proposed, which provides an empirical basis for breaking the administrative boundary barriers and has strong practical significance. In conclusion, this study not only has innovative perspectives in theory, but also provides important insights for regional coordinated development in practice.

**The interesting and notable findings of this study compared to existing research may include:** (1) The daily population flow intensity between developed cities is much higher than during the Spring Festival. For example, the average daily mobility intensity between Shanghai and Suzhou on weekdays is 33% higher than during the Spring Festival. This challenges the traditional understanding in classical population mobility models that "festival homecoming tides dominate population mobility intensity", and suggests that the driving force behind population mobility in the Jiangsu-Zhejiang-Shanghai region of China has shifted from "periodic festivals" to "normalized market demands". (2) Results from disruption scenario simulations show that networks based on core nodes are efficient but have high vulnerability. This provides a quantitative tool for urban risk prevention and enriches the analysis of disaster chain networks. (3) Administrative boundaries have a stronger inhibiting effect on links between small and medium-sized cities than between core cities, extending existing discussions on the impact of administrative divisions. It also proposes a "resilience-oriented" regional policy design pathway that provides empirical evidence for breaking down administrative boundary barriers. (4) Detailed discussions and summaries of the above findings are presented in "Section 4 Discussion" and "Section 5 Conclusions".

**The reviewer's comment "The static characteristics of network resilience in natural scenarios were analyzed"** mainly refers to the third research result in the abstract. To improve clarity and rigor, we have optimized the language in the Abstract section. In addition, "analyzing the static characteristics of network resilience in natural scenarios" involves selecting four indicators - centrality, hierarchy, agglomeration, and transportability - by reviewing, comparing, and summarizing core metrics from the classical literature to analyze network characteristics. Guided by the research framework, this analysis systematically examines the importance of nodes in the network, the hierarchical and redundant nature of the division of labor system, the tightness of connections within subgroups, and the flow efficiency of network elements. Thus, we study the static characteristics of the network under normal conditions-i.e., the functional structural features and operational patterns of the urban network provide clues for optimizing regional development layouts. Specific conceptual definitions and research methods can be found in **"(1) Normal scenario: static characteristics analysis of network resilience" within "2.4.2 Methods of scenario simulation"**, and the research results are detailed in **"3.2.1 Analysis of network structure resilience under normal scenario"**.

*2. There are some formatting problems in the reference.*

**The author’s answer:** Thank you for your valuable suggestions. According to the Submission Guidelines of *PLOS One*, we used the latest style file "PLoS.ens" provided by EndNote to format the references after carefully reviewing the content of the article.

*3. There are some language specification problems, for example, in line 171, the terms “betweenness centrality’ and “closeness centrality” seem to be more recognized than “mediating centrality” and “proximity centrality” used in this article.*

**The author’s answer:** Thank you very much for your valuable comments. According to your suggestions and the authoritative references, the relevant terms in the manuscript have been **expressed as “betweenness centrality” and “closeness centrality”** by the norms of academic terminology. We have also double-checked and revised the expression of other specialized terms in the manuscript.

*4. There are inconsistencies in the use of the same term, e.g., whether “toughness” or “resilience’ is used for resilience?*

**The author’s answer:** Your constructive feedback is greatly appreciated. Based on the study’s core framework and authoritative academic literature, the manuscript has standardized the expression of relevant concepts under academic terminology norms. Specifically, **this study uniformly uses "resilience"**, which accurately summarizes the resilience, adaptive capacity, and transformational potential of urban networks from the perspective of dynamic systems theory. In contrast, “resilience” is more in line with the theoretical core and analytical dimensions of this study than “resilience”.

*5. In the part 3.2.1, line 353: What is “compose centrality”? What is the basis for normalizing the three centrality indicators to obtain the so-called “compatible centrality”? The authors should provide some supporting evidence, such as references. Also, in the caption of Figure 5, “compose centrality” is written as “integrated centrality”.*

**The author’s answer:** We sincerely appreciate your valuable suggestions. There may have been differences in linguistic translation in the previous expression. Based on the core meaning of this indicator and after consulting authoritative references, we have **standardized the terminology as "composite centrality"** and cited the relevant literature in the text. For details, please refer to **"2.4.3 Methods of measuring the network structural resilience"** in the article, where the last paragraph of **"(1) Centrality"** elaborates on the connotation, role, and calculation method of "composite centrality."

**Conceptual connotation:** Composite centrality comprehensively reflects a node’s breadth of connections, hub control capacity, and information transmission efficiency. This study comprehensively applies degree centrality, betweenness centrality, and closeness centrality to construct the "composite centrality", providing a multi-dimensional perspective to fully characterize the role of cities. It helps to avoid evaluation biases caused by single-dimensional centrality indicators. For example, it can meticulously identify "high-impact but low-connectivity" potentially vulnerable nodes.

**Calculation method:** This study first uses z-score standardization to eliminate dimensional differences among the three indicators (degree centrality, betweenness centrality, and closeness centrality). It then applies the entropy weight method to calculate their weight coefficients, thereby deriving the composite centrality for each node. Correspondingly, we have revised the legend of Figure 5 to standardize the terminology for "betweenness centrality," "closeness centrality," and "composite centrality." We have also carefully reviewed the content of all other figures in the article.

**Response to Reviewer #3**

*The paper explores the spatiotemporal dynamics of population flow and network resilience within the Jiangsu-Zhejiang-Shanghai region using one-year Baidu daily migration scale index datasets from 2023. The topic is very interesting. There are some issues that need to be addressed.
1. The data used in the manuscript is the daily migration scale index rather than OD flows. How is the index calculated and what does its value mean?*

**The author’s answer:** Thanks for your valuable suggestions. The data used in this study are sourced from Baidu Migration (https://qianxi.baidu.com), whose migration index is a relative intensity indicator generated from anonymized user geofencing data based on LBS signalling. **As a standardized metric of origin-destination (OD) volume, the migration index exhibits a strong correlation with actual OD traffic and better captures the relative intensity or patterns of migration behavior**. In this paper, the average daily migration scale index—calculated as the ratio of the total migration scale index between two cities over a specific period to the number of days in that period—is used to represent the "average daily connection intensity," characterizing the population mobility pattern during that period. For details, please **see section "2.4.1 Method of network construction"**.

**Calculation method:** According to the complex network theory, cities are considered as nodes, and population migration interactions between cities are modelled as weighted edges. Leveraging daily datasets from the Baidu Migration Platform—including daily total migration index (***T)*** (See the table below for examples of data), origin-destination pairs, and directional migration proportions (***I)*** — we constructed 365 daily 25×25 connection matrices to represent the population flow network among 25 cities over a year. The daily average migration scale index, $\boldsymbol{T}_{\boldsymbol{day}}$ is calculated by averaging the bidirectional connection strength between cities over a specified period, reflecting the intensity of population flow interactions. Higher $T_{day}$ values signify intensified population mobility interdependence between city pairs. The formula is:

$$\begin{aligned} \boldsymbol{T}_{\boldsymbol{day}（\boldsymbol{i},\boldsymbol{j}）}={\frac{\mathbf{1}}{\boldsymbol{D}}\sum_{\boldsymbol{d}=\boldsymbol{1}}^{\boldsymbol{D}} \boldsymbol{(}\boldsymbol{T}}_{\boldsymbol{i}}\boldsymbol{\times}\boldsymbol{I}_{\boldsymbol{ij}}+\boldsymbol{T}_{\boldsymbol{j}}\times\boldsymbol{I}_{\boldsymbol{ji}})\boldsymbol{\#} \end{aligned}$$

where D denotes the number of days in a given period. $T_{i}$ and $T_{j}$ are the migration indices of cities i and j, respectively. $I_{ij}$ denotes the proportion of migration from city i to city j, and $I_{ji}$ denotes the proportion of migration from city j to city i.

Table. Example of Processed Baidu Migration Big Data

(Full dataset is available on the Baidu Migration Platform-https://qianxi.baidu.com)

| **Date** | **City** | **Migration Index (*T*)** | **Destination City** | **Type** | **Migration Proportion (*I*)** |
| --- | --- | --- | --- | --- | --- |
| 20230101 | Hangzhou | 5.5251072 | Jiaxing | Outbound | 13.87% |
| 20230101 | Hangzhou | 5.5251072 | Shaoxing | Outbound | 11.90% |
| 20230101 | Jinhua | 1.3152132 | Hangzhou | Inbound | 22.15% |
| 20230101 | Jinhua | 1.3152132 | Lishui | Inbound | 7.04% |

*2. In Section 2.4.2, do the authors consider travel time or other impacts that reflect the level of transportation facilities when measuring network transmission efficiency? If not, please explain the reason.*

**The author’s answer:** We extend our heartfelt gratitude for your thorough review and insightful comments. This study **did not consider the impact of travel time or transportation infrastructure** when measuring network transmission efficiency.

**The reasons are as follows:** The "network transmission efficiency" selected in this paper is **a targeted indicator with topological properties designed to measure network resilience**. Network efficiency E (where 0≤E≤1) quantifies transmission efficiency. Higher E values indicate greater network efficiency and stronger flow and transmissibility of resources and people within the network. Since "network transmission efficiency" **is not a mathematical metric directly tied to time or speed,** the above factors were not incorporated into the analysis.

*3. In Section 3.1.2, the authors just compared the differences in migration index between the spring festival and a typical month. Given the advantage of having a full year of data, a more detailed exploration of temporal flow variations would be beneficial. Besides, the results in Figure 4 are not very clear.*

**The author’s answer:** Your constructive feedback is greatly appreciated. **The primary objective of this study is to reveal the response mechanisms of urban network resilience** under different scenarios by analyzing the structural and resilience characteristics of population mobility networks, and to propose improvement strategies for identified core nodes and critical vulnerable links to inform regional spatial coordination and sustainable development. Based on this, Section 3.1 elaborates on the spatiotemporal characteristics of population mobility networks, including: (1) Characterising the overall annual network characteristics by calculating the average daily migration intensity from 365 days of big data throughout the year. (2) Intercepting data from the Spring Festival period and normal daily periods to compare and analyze differences in network characteristics across typical periods. **By depicting both annual and typical population mobility networks**, the above analyses provided a comprehensive and solid foundation for the subsequent network resilience analysis. **Given that the main focus of the study is on urban network resilience**, rather than simply discussing population mobility characteristics - and due to space constraints in the paper - it is not appropriate to further subdivide further periods to analyze changes in population mobility. Your suggestion to explore the temporal variations in population mobility in more detail is very meaningful and will serve as an important direction for our future in-depth research.

**In response to your comment that the results in Figure 4 were not clear enough,** we have optimized the color scheme, styling, and annotations in the figure after reviewing the relevant content. We have also polished the linguistic logic and grammar of the corresponding paragraphs in the manuscript. Additionally, we have improved the presentation of other figures in the study and their accompanying text. We hope this allows us to convey the study’s key information comprehensively and clearly.

*4. Regarding the scenario simulation in Section 3.2.1, how are random and deliberate perturbations defined and implemented?*

**The author’s answer:** Thank you very much for your question. We sincerely apologize for the insufficient specificity in our previous description of the perturbation scenarios. Based on the research context and field conventions, we have **revised the terminology: "random perturbation" is now expressed as "Random Disruption", and "deliberate perturbation" is now "Targeted Disruption"** to ensure consistency throughout the paper and avoid ambiguity.

In the revised manuscript, Section "2.4 Methods" has been expanded with a new sub-section, "2.4.2 Methods of scenario simulation", which outlines the logic for simulating normal and disruption scenarios. Within this section, **"(2) Disruption scenario: dynamic characteristics analysis of network resilience" provides a detailed explanation** of the conceptual connotations, implementation methods, measurement indicators, and roles of random and targeted disruptions. The following table offers a brief summary of random and targeted disruptions under disruption scenarios, with **full details available in part (2) of Section 2.4.2.**

Table. Brief description of random and targeted disruptions

| **Disruption scenario** | **Academic definition** | **Scientific significance** | **Real-world example** |
| --- | --- | --- | --- |
| Random disruption | Randomly remove network nodes and associated edges with uniform probability to simulate unpredictable sudden shocks. | Evaluate the resilience change of networks to random failures. | Random interruption of road traffic caused by an earthquake. |
| Targeted disruption | Remove nodes and associated edges in descending order of importance to simulate intentional attacks. | Identify core network nodes and cascading failure risks. | Precision strikes on hub cities during wartime. |

*5. There are some grammar errors and long sentences such as in Lines 77-82. The authors are recommended to revise the text to improve clarity and readability.*

**The author’s answer:** We sincerely appreciate your thorough proofreading and valuable suggestions. We sincerely apologize for the inadequate grammatical expressions in our earlier draft. The sentences in lines 77–82 of the first draft have been carefully reviewed and revised. Additionally, we have conducted a meticulous proofread of the entire manuscript to improve grammar and polish the language, striving to present the research more rigorously and scientifically to editors, reviewers, and readers alike.
