# Supplementary material for: Exploring the spatiotemporal dynamics and resilience assessment of urban networks from the perspective of population flow
Source: PLoS One. 2025 Jun 18;20(6):e0325908. doi: 10.1371/journal.pone.0325908 (PMC12176158; doi:10.1371/journal.pone.0325908)
Supplement: Attachment — Submitted filename: Response_to_Reviewers_auresp_2.docx [file pone.0325908.s003.docx]

**Response Letter**

Dear Editors and Reviewers:

We sincerely appreciate your insightful comments and professional suggestions on our manuscript entitled “*Exploring the Spatiotemporal Dynamics and Resilience Assessment of Urban Networks from the Perspective of Population Flow*” (Manuscript Number: PONE-D-25-01272). Your invaluable feedback has not only significantly improved the quality of this manuscript but also provided important guidance for our ongoing research. In response to your comments, we have undertaken comprehensive revisions to the manuscript, which include but are not limited to:

1. Standardized section numbering and verified figure accuracy.

2. Refined Sections 4.2 and 4.3 to enhance clarity and rigor.

3. Reviewed and updated “References” to comply with journal guidelines.

The following section provides a point-by-point response to each of your specific comments. We believe these revisions have strengthened the manuscript substantially, and we are grateful for your time and expertise. Please do not hesitate to contact us if you require any additional information or clarification.

Yours sincerely,

Jiulin Li，Wenhui Lin，Jinlong Chu

**Response to the comments**

*Thank you for your thorough revisions and for addressing the reviewer's comments in detail. Upon reviewing the revised manuscript, I appreciate the many positive and effective improvements you have made, which have significantly enhanced the quality of the paper. I especially commend your efforts in clarifying the methodological details and terminology, which have further strengthened the scientific rigor and readability of the manuscript.*

*However, after another review, I noticed the following points that need further attention and clarification:*

*1. In Figures 3, 4, and 7, Taizou (JS) is positioned at the bottom, and Taizhou (ZJ) is at the top. However, in Figures 1 and 5, Taizhou (JS) is at the top, and Taizhou (ZJ) is at the bottom. This inconsistency in figure layout should be addressed. I recommend ensuring uniformity in the positioning of the cities across all figures.*

**The author’s answer:** Thank you for bringing this important issue to our attention. We apologize for the inconsistency in the figure layout. We have re-examined the position labels of all the figures to ensure consistency in the geographical location of the cities. **The layout of Figures 3, 4, and 7 has been revised to show Taizhou (JS) at the top and Taizhou (ZJ) at the bottom, consistent with Figures 1 and 5.** We paid special attention to the standardization and consistency of the study, and also carefully checked the content of the other figures in the manuscript. This revision ensures that all spatial information is conveyed accurately. We hope that the information covered in the study has been fully communicated to you.

*2. I noticed that two sections are titled “3.2.1.” However, there is no section 3.2.2 in Chapter 3.2. Please check if the numbering should be revised or if a section is missing, and adjust the titles accordingly.*

**The author’s answer:** Thank you for your valuable comments. The section numbering issue you pointed out is important. Upon reviewing the section structure of the entire text, we discovered a duplication of numbering in section 3.2 of the original draft. Therefore, we have **changed the second subsection from "3.2.1" to "3.2.2".** We have reorganized the numbering system of section 3 to ensure that the subsequent levels correspond to each other. These revisions have clarified and rationalized the chapter structure of the thesis. Thank you for your help in improving the organization and readability of the thesis.

*3. It is unclear whether the resilience calculation is based on daily population flow data or on Spring Festival population flow data. Since you emphasized the importance of both states in the static analysis, and considering that both may face different disruptions, it would be beneficial to discuss resilience in the context of both population flow states.*

**The author’s answer:** Thank you very much for your valuable suggestions, which are an important inspiration for the study. Regarding the use of **annual daily average population flow data as the basis for resilience calculation**, we make the following clarifications:

**(1) Data selection and processing:**

**Urban network resilience is a structural characteristic formed by the long-term evolution of a region.** The attributes it reflects, such as the hierarchy of network division of labor, the closeness of subgroup connections, and the effectiveness of factor flow, are the results of mutual interactions of the urban network system in the process of long-term development. Therefore, the selection of annual daily average data (covering 365 days) can effectively avoid the interference of short-term fluctuations in specific periods, such as the Spring Festival (14 days) and the daily period (30 days in November, for example), on the analysis results, so as to reflect the level of urban network resilience more objectively.

**(2) Specific analysis process:**

①This study analyzes population flow throughout the year, the Spring Festival, and the daily period. It unifies the data comparison benchmarks in different periods by standardizing daily averages. This allows for a deeper exploration of the spatio-temporal characteristics of the urban network. ②**Using annual daily average population flow data as the core basis**, we reveal the static characteristics of network resilience in normal scenarios and disruption scenarios through quantitative analysis. ③Based on the spatio-temporal patterns and resilience characteristics of the population mobility network, we propose policy recommendations.

**(3) Expansion and reflection of research direction:**

Your suggestion to explore differences in resilience characteristics in different periods is meaningful and will be an important direction for future research. Due to the limited length of the article, it is difficult for this study to break down periods further to explore resilience in depth. In follow-up research, we plan to develop a dynamic resilience assessment framework based on long time series, which will **focus on analyzing resilience performance in the context of extreme flows, such as special periods and events. The revised manuscript of the current study provides additional explanation in section "4.3 Limitations and future directions".**

Thank you again for your valuable suggestions, which will greatly help us improve the current study and guide subsequent research.

*4. In Section 4.2.2, you suggest that "Lianyungang and Yancheng should focus on upgrading tourism infrastructure and service ecosystems to promote livability-driven growth," among other recommendations. However, these insights do not seem to be directly derived from the analysis of population flow. Could you please provide a clearer explanation of how these industry recommendations are linked to the population flow analysis?*

**The author’s answer:** Thank you very much for your valuable comments. We are keenly aware of the lack of logical rigor in our previous policy recommendations. Regarding the connection between the recommendations and the population mobility network analysis, we offer the following clarifications:

**(1) The basis of the policy recommendations:**

①Population flow is usually a rational choice based on multidimensional considerations, such as the level of urban development, industrial economic benefits, supporting facilities and services, and regional culture. This study constructs a city network based on population flow and analyzes its spatial-temporal characteristics and resilience level to accurately identify city nodes with weak population attraction, a lack of development momentum, and lower resilience. **Combining the results of the study, the current state of urban development and relevant planning documents**, we propose suggestions to improve the urban network system in the study area. This will enhance the region's overall development quality and risk-resistant capacity. ②**Industry is one of the key factors affecting population mobility and urban development.** The results of the network resilience assessment show that northern Jiangsu and southern Zhejiang (including Lianyungang, Suqian, and Quzhou, among other cities) have obvious obstacles in the "core-edge" connection. This results in the region's comprehensive competitiveness being much lower than that of central, developed areas. Despite their unique location advantages and rich ecological and cultural resources, these regions have not realized synergies with developed regions due to their lack of related industries. Therefore, **this study considers industry as an important perspective in the policy recommendations.**

**(2) Revised manuscript description:**

①We have systematically optimized the logic and presentation of Section 4.2.2. We have strengthened the linkage with network resilience, and put forward policy recommendations that are more in line with the findings of the study and the reality of the city. Specifically, we added the direct correspondence between network resilience and population mobility recommendations. **We emphasize the significance of developing cultural and tourism industries based on location and resource endowment, and upgrading supporting services, for small and medium-sized cities to attract population flows and promote the dynamic equilibrium of regional mobility networks.** ②We have also optimized the elaboration of other policy recommendations in Section 4.2, aiming to convey the core ideas of the study more clearly and accurately.

Thank you again for your help in identifying the shortcomings of our argument. Your feedback is crucial to enhancing the rigor and academic value of this study.

*Thank you once again for your efforts in improving the manuscript. I believe that addressing these issues will further strengthen the rigor and academic value of the paper.*

**The author’s answer:** We sincerely thank you for your careful review and valuable comments on this study. We have responded to the review comments one by one and revised the relevant contents. And we have done a deep reflection on our scientific rigor. These changes will greatly benefit our future scientific research.
